# Within-host SARS-CoV-2 diversity in immunocompromised patients during acute infection

**DOI:** 10.1128/jvi.02224-25

**Published:** 2026-06-09

**Authors:** Deninson Alejandro Vargas, Ludwig L. Albornoz, Mateo Peña-Morales, Helen Johana Ortiz Rojas, Mallery I. Breban, Nathan D. Grubaugh, Anne M. Hahn

**Affiliations:** 1 Centro Internacional de Entrenamiento e Investigaciones Médicas (CIDEIM), Cali, Colombia; 2 Universidad Icesi 28012, Cali, Colombia; 3 Departamento de Patología y Medicina de Laboratorio, Fundación Valle del Lili 67597, Cali, Colombia; 4 Clinical Investigation Group, Universidad del Rosario 25807, Bogotá, Colombia; 5 Hospital Universitario Mayor Méderi, Bogotá, Colombia; 6 Centro de Investigaciones Clínicas, Fundación Valle del Lili 655490 , Cali, Colombia; 7 Department of Epidemiology of Microbial Diseases, Yale School of Public Health 50296 , New Haven, Connecticut, USA; Fred Hutchinson Cancer Center Vaccine and Infectious Disease Division, Seattle, Washington, USA

**Keywords:** intra-host diversity, SARS-CoV-2, immunocompromised, acute phase

## Abstract

Within-host SARS-CoV-2 diversity during acute infection is largely characterized by low-frequency intra-host single-nucleotide variants (iSNVs), but whether early iSNV patterns differ by immune status remains unclear. We analyzed biobanked diagnostic specimens from adults with RT-qPCR-confirmed SARS-CoV-2 infection collected in Cali, Colombia, between 2020 and 2022. Immunocompromised cases sampled during the acute phase were classified into four clinical categories, and immunocompetent cases from the same catchment area were used for comparison. Whole-genome sequencing (Illumina) yielded 102 high-quality genomes (≥85% coverage; immunocompetent, *n* = 60; immunocompromised, *n* = 42) from 114 samples. iSNVs were identified using iVar with stringent thresholds (depth ≥400× , Phred ≥20, allele frequency 3%–97%). Primary outcomes included iSNV counts, allele-frequency distributions, within-host complexity (Shannon entropy), normalized polymorphic sites, and Hamming distance. Analyses were conducted across all circulating lineages and within the Mµ (B.1.621) variant. Across all lineages, normalized polymorphic sites differed by immune status, with higher values in immunocompetent individuals (*P* = 0.0064), whereas iSNV counts, allele-frequency distributions, and Shannon entropy were comparable between groups. When restricted to Mµ, immunocompetent individuals exhibited higher iSNV allele frequencies in the Spike region (*P* = 0.0016). Within Mµ, mutational profiles differed by immune status: S-region iSNVs were predominantly synonymous in immunocompromised individuals and non-synonymous in immunocompetent individuals. Recurrent iSNVs revealed a shared core mutational signature across immune groups, with no Mµ-specific recurrent iSNVs exclusive to immunocompromised patients. Overall, intra-host diversity during acute infection was broadly similar by immune status, while lineage- and region-specific signals in the S region highlight nuanced early sub-consensus dynamics warranting functional investigation in immunophenotype-informed studies.

**IMPORTANCE:**

Understanding how immune status shapes within-host SARS-CoV-2 diversity is key for interpreting transmission risk and evolutionary potential. Immunocompromised patients are often presumed to harbor more diverse viral populations, yet evidence early in infection remains limited. Analyzing samples, we compared minor variant (intra-host single-nucleotide variant [iSNV]) patterns between immunocompromised and immunocompetent individuals and evaluated lineage-specific effects. We found comparable within-host diversity, but normalized polymorphic sites were higher in immunocompetent than in immunocompromised individuals when considering all lineages. We observed an Mµ-restricted S-region signal: immunocompetent hosts showed higher S-region iSNV allele frequencies, whereas immunocompromised hosts exhibited a shift toward synonymous S-region iSNVs. These results refine assumptions about early infection in immunocompromised hosts, emphasize the value of reporting synonymous and non-synonymous changes, and highlight that conclusions can depend on viral lineage and the diversity metric considered. Our study underscores the need for immunophenotype-informed studies to test whether synonymous iSNVs modulate viral expression, replication, and transmission.

## INTRODUCTION

Within-host genetic diversity during acute SARS-CoV-2 infection is characterized by low-frequency levels of intra-host single-nucleotide variants (iSNVs) when viral loads are high early in the infection (1–3). Only a few iSNVs increase in frequency and are successfully transmitted to other hosts, eventually becoming fixed in the population (4, 5). A key factor determining the likelihood of a new iSNV becoming fixed is the transmission bottleneck, which is defined as the number of viral particles transmitted from a donor to a recipient host that successfully establish genetic lineages within the recipient (6), usually estimated to range between one and three viral particles (2, 7, 8), and suggests that iSNVs are likely to be lost during transmission.

Despite the low frequency of emerging SARS-CoV-2 iSNVs, specific mutations that confer enhanced transmission or immune evasion may spread rapidly if successfully transmitted (2). A previous study demonstrated that phylogenetic changes were strongly associated with high-confidence iSNVs, consistent with fixation after transmission or *de novo* mutations reaching consensus. Interestingly, shared iSNVs that never reached consensus were not reflected in the phylogenetic tree analysis (2). However, iSNVs that are successfully transmitted and later emerge in variants of interest (VOIs) or concern (VOCs) are typically reported only after their characteristic mutations reach an intra-host frequency greater than 50%. Thus, these mutations may be detectable at lower frequencies within the host long before VOIs or VOCs are identified (4).

In large population studies, iSNVs have been detected in up to 27% of cases (9). However, within-host viral genetic diversity can vary with viral load (for which RT-qPCR cycle threshold (Ct) serves as an inverse proxy), alongside age and immune status following natural infection or vaccination. Regarding vaccination, evidence is mixed: some reports describe higher within-host diversity among vaccinated individuals (5, 10), whereas others find reduced diversity in vaccinated individuals (10–12), with mutations enriched in the Spike region (11). These findings suggest that vaccine-associated effects on within-host diversity are context-dependent, varying by viral lineage, vaccine platform, and the level of protection achieved.

The emergence of new, genetically divergent SARS-CoV-2 variants has been associated with chronic or prolonged infections in immunocompromised individuals. Several studies have shown that patients with various forms of immunocompromise, such as solid tumors, hematological malignancies (13–17), severe antiphospholipid syndrome (18), as well as solid organ transplant recipients (19), exhibit increased SARS-CoV-2 genetic diversity within the host or mutations related to immune evasion. However, none of these studies focused on assessing differences during the acute phase of infection or on comparing viral evolution in immunocompetent versus immunocompromised individuals.

In this study, we characterized intrahost diversity and compared between-host patterns in immunocompromised versus immunocompetent patients sampled during the acute phase of SARS-CoV-2 infection in Cali, Colombia (2020–2022). Immunocompetent cases were selected from the same catchment area and sampling period as the immunocompromised cases. Our primary objective was to determine whether early iSNV diversity differs by immune status and, secondarily, to assess whether these patterns vary by viral lineage and patient-level factors, and whether some iSNV changes persist over time across different viral lineages.

## MATERIALS AND METHODS

### Study participants

A total of 114 residual clinical samples obtained during routine care (2020–2022) were included: 96 nasopharyngeal swab samples, 15 nasopharyngeal aspirate samples, 2 tracheal secretion samples, and 1 extracted RNA sample. All specimens were stored in the research biorepository of Fundación Valle del Lili Clinical Research Center. This retrospective group included adults (≥18 years) with RT-qPCR-confirmed SARS-CoV-2 infection. Immunocompetent cases were selected from the same catchment area and sampling period as the immunocompromised cases. Immunocompromised participants were classified into four categories: (i) hematologic malignancies, (ii) malignant solid tumors, (iii) hematopoietic progenitor or solid-organ transplantation, and (iv) primary or secondary immunodeficiencies or autoimmune diseases (hereafter referred to as immunodeficiencies or autoimmune diseases). Clinical variables were extracted from participants’ medical records (Table S1 and Fig. S1), and available treatment information is reported in Table S2.

### Viral RNA extraction

Banked specimens were stored at −80°C until processing. SARS-CoV-2 RNA was extracted using the GeneJET Viral DNA/RNA Purification Kit (Thermo Fisher Scientific) according to the manufacturer’s protocol. Briefly, 200 µL of sample was mixed with 200 µL lysis buffer, 5 µL RNA carrier, and 50 µL proteinase K; nucleic acids were precipitated with 300 µL absolute ethanol, washed sequentially with 500 µL wash buffer 1 and 700 µL wash buffer 2, and eluted in 30 µL elution buffer. Extracts were stored at −80°C until real-time RT-PCR.

### RT-qPCR for SARS-CoV-2

SARS-CoV-2 was detected using the VIASURE Real-Time PCR Detection Kit (CerTest Biotec) per the manufacturer’s instructions. The assay targets ORF1ab and N. Each reaction was prepared with 15 µL rehydration buffer and 5 µL RNA template, sealed, and briefly centrifuged (~10 s). Amplification was performed on a CFX96 Touch Real-Time PCR System (Bio-Rad) with the following cycling: reverse transcription at 45°C for 15 min; initial denaturation at 95°C for 2 min; 45 cycles of 95°C for 10 s and 60°C for 50 s. Samples with a Ct ≤36 were retained for sequencing.

### Library preparation and sequencing

The Illumina COVIDSeq Test RUO version was used to generate whole genome sequencing libraries for samples with ORF1ab and N PCR mean Ct values ≤36. The manufacturer’s protocol was slightly modified by replacing the manufacturer’s primers with the ARTIC V4.1 primer pools (Integrated DNA Technologies) (https://github.com/artic-network/artic-ncov2019/tree/master/primer_schemes/nCoV-2019) and lowering the annealing temperature to 63°C during amplicon generation, and by shortening the tagmentation step to 3 min. Final libraries were pooled, cleaned, and quantified using the Qubit High Sensitivity 1× dsDNA Assay Kit (Life Technologies). The libraries were deep-sequenced using 2 × 150 bp paired-end reads on an Illumina NovaSeq 6000 at the Yale Center for Genome Analysis. For each sample, at least one million paired-end reads were generated. To monitor for cross-contamination, we included three non-template controls, which were added at RNA extraction, cDNA synthesis, and amplicon generation steps, with each sequencing batch. We ensured that no or fewer than 100 SARS-CoV-2 reads were generated in each control before proceeding with the analysis.

The sequencing data were demultiplexed and processed, including conversion from base call (BCL) to FASTQ format and adapter trimming, using the Illumina bcl2fastq pipeline (v.2.20.0). To generate consensus SARS-CoV-2 whole genomes, reads were aligned to the ancestral SARS-CoV-2 reference genome (GenBank accession number MN908947.3) using BWA-MEM (20) (version 0.7.15) to produce indexed and sorted binary alignment map (BAM) files. Adapters were trimmed, primers masked, and consensus base calls generated from the BAM files using a simple majority (>60% base frequency) with iVar (21) (version 1.3.1) and SAMtools (22) (version 1.7). Ambiguous base calls were defined as nucleotide sites containing fewer than 20 uniquely mapped reads. To validate the sequencing runs, negative controls were sequenced, which in all cases consisted of >99% sites with Ns. We selected sequences containing >70% non-N base calls for submission to GISAID. SARS-CoV-2 lineages were assigned using Pangolin (23) (version 3.1.17). Maximum-likelihood phylogenies of the consensus genomes were further visualized using the Nextstrain augur pipeline (24).

### iSNV identification

Samples with >1,000,000 total reads and an average depth ≥400× were retained. Corresponding BAM files were processed with iVar v1.3.1 (21) to call iSNVs using the following thresholds: per-site depth ≥400 reads, Phred quality ≥20, and allele frequency 0.03–0.97. The iVar output was then summarized to obtain the total iSNV count per sample and to annotate variant attributes for downstream analyses.

### Sample size

The planned sample size was estimated assuming a 23 percentage-point difference between groups (15% in immunocompetent controls vs 38% in immunocompromised patients), based on the proportions reported by Siqueira et al. (13). With 80% power, the required sample size was 57 patients per group, for a total of 114 participants. Although this was the original target, the final analytic sample included 102 patients: 60 immunocompetent and 42 immunocompromised.

### Statistical analysis

Analyses were performed in R v.4.2.3. Outcomes included: iSNV count per genome, iSNV allele-frequency (AF) distributions, within-host complexity (Shannon entropy), the number of unique polymorphic sites normalized by mean sequencing depth, and Hamming distance (25). Because the final sample was smaller than planned and the groups were unequal in size, between-group comparisons were performed using nonparametric methods. Comparisons between immunocompetent and immunocompromised patients used Wilcoxon rank-sum tests for iSNV counts, Shannon entropy, and region-level AF summaries. Effect sizes were reported as Cliff’s δ (26) with 95% CIs. Associations between iSNV counts and age, days from symptom onset, and Ct values were assessed using Spearman’s rank correlation coefficient. Differences by sex were also evaluated using the Wilcoxon rank-sum test and Cliff’s δ. *P*-values <0.05 were considered statistically significant.

## RESULTS

### Demographic and clinical characteristics of the study population

**Fig 1:**
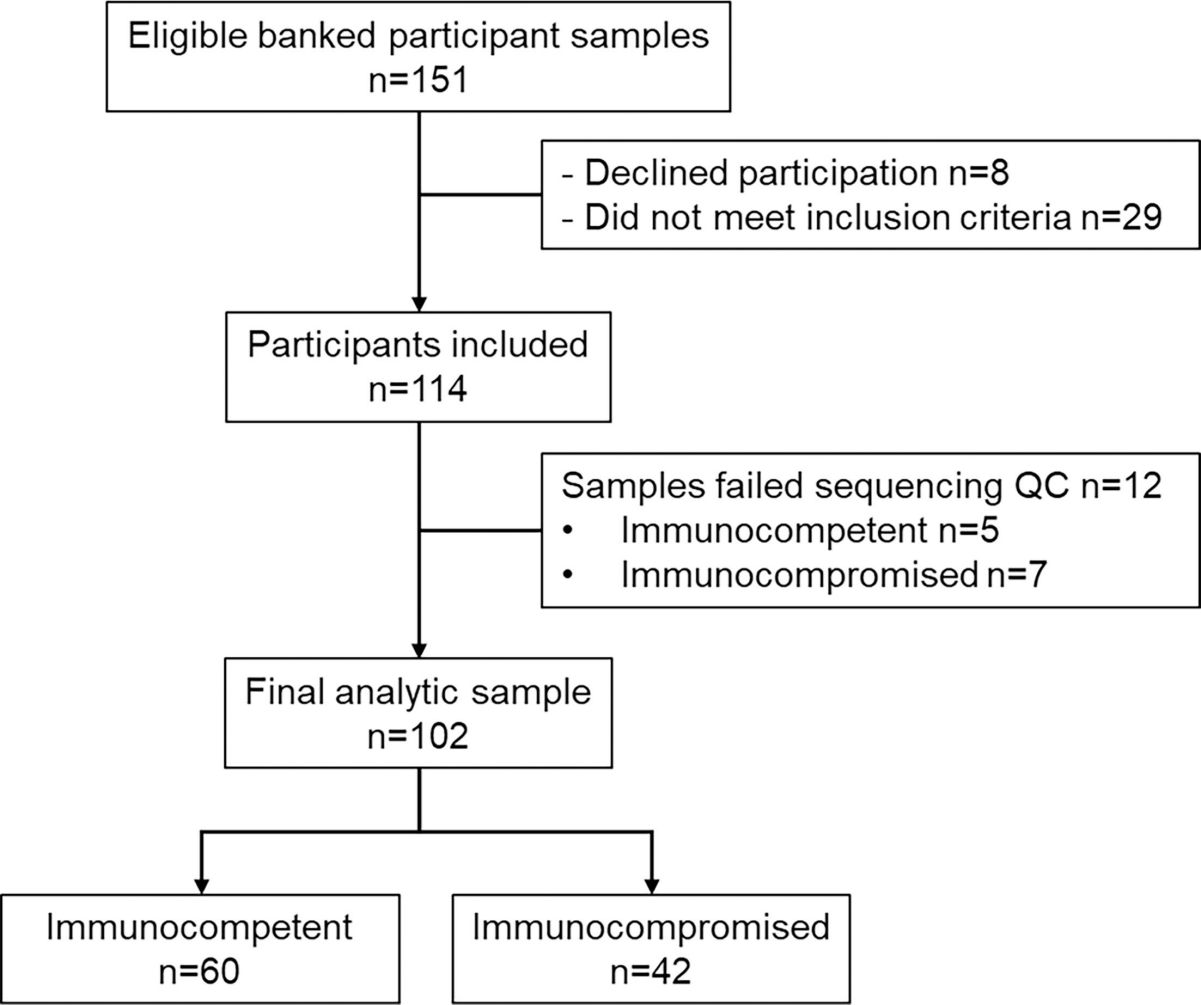
Flow diagram of participant screening, inclusion, and final analytic sample.

A total of 114 participants were included, each contributing one acute respiratory sample, from 151 participants initially screened. Whole-genome SARS-CoV-2 sequencing was attempted directly from these specimens using a tiled-amplicon Illumina approach (2 × 150 bp paired-end reads). Samples were obtained during routine care between 2020 and 2022 and included 96 nasopharyngeal swabs, 15 nasopharyngeal aspirates, 2 tracheal secretion samples, and 1 extracted RNA sample. Thirty-seven participants were excluded because they did not meet the inclusion criteria or declined to participate (Fig. 1). In addition, 12 samples failed sequencing quality control (seven from the immunocompromised group and five from the immunocompetent group). High-quality, high-coverage genomes (≥85% coverage) were recovered from 102/114 samples included in the final analysis.

Among included participants, 64/102 (62.7%) were female. The median age was 44 years, and the median time from symptom onset to sampling was 3 days (Table 1). Total iSNV counts did not differ by immune status (Wilcoxon rank-sum, *P* = 0.381; Cliff’s δ 0.10, 95% CI −0.13 to 0.33, classified as negligible (|δ| <0.15), or across immunocompromised subgroup categories (Fig. S1). No statistically significant association was detected between iSNV counts and age (Spearman ρ = −0.079, *P* = 0.434) or days from symptom onset (ρ = −0.109, *P* = 0.292). Results were similar by sex (Wilcoxon *P* = 0.394), with a small effect size (Cliff’s δ = −0.10; 95% CI −0.32 to 0.13).

**TABLE 1:** Clinical and demographic characteristics^*c*^

Characteristic	Immunocompetent participants (*n* = 60)	Immunocompromised participants (*n* = 42)	Overall (*n* = 102)	*P*
Female, *n* (%)	44 (73.3)	20 (47.6)	64 (62.7)	0.015^*a*^
Age, years, median [IQR]^*d*^	39.5 (29.7–57.0)	55.0 (39.2–72.7)	44.0 [32.0–64.7]	0.002^*b*^
Days from symptoms onset, median [IQR]	2.00 [1.0–4.0]	5.0 [2.0–7.0]	3.0 [1.00–7.00]	0.010^*b*^
Ct value, median [IQR]	16.2 [4.0–22.0]	17.7 [15.4–20.5]	16.8 [14.5–20.8]	–^*e*^
Vaccinated (≥1 dose), *n* (%)	45 (75.0)	27 (64.3)	72 (70.6)	–
COVID-19 severity, *n* (%)				–
Mild	45 (75.0)	20 (47.6)	65 (63.7)	
Moderate	4 (6.7)	5 (11.9)	9 (8.8)	
Severe	3 (5.0)	3 (7.1)	6 (5.9)	
Critical	5 (8.3)	3 (7.1)	8 (7.8)	
Not reported	3 (5.0)	11 (26.3)	14 (13.8)	
Type of immunocompromise				
Hematologic cancer, *n* (%)	–	8 (19.0)	8 (7.8)	
Solid tumor, *n* (%)	–	20 (47.6.0)	20 (19.6)	
Hematopoietic or solid organ transplantation, *n* (%)	–	8 (19)	8 (7.8)	
Immunodeficiencies or autoimmune diseases, *n* (%)	–	8 (19.0)	8 (7.8)	
Discharge status alive, *n* (%)	55 (91.7)	37 (88.1)	92 (90.2)	–

^*a*^ *P*-values: Fisher’s exact test.

^*b*^ *P*-values: Mann-Whitney *U*-test.

^*c*^ Some patients may be represented in more than one category because they presented with more than one condition.

^*d*^ IQR, interquartile range.

^*e*^ –, no statistically significant difference.

Within the immunocompromised group, the most frequent category of immunocompromise was solid tumor (20/42, 47%), followed by the other three categories, each representing 19% of cases (8/42) (Table 1).

### Mµ (B.1.621) predominance and immune status-associated differences in polymorphic sites

**Fig 2:**
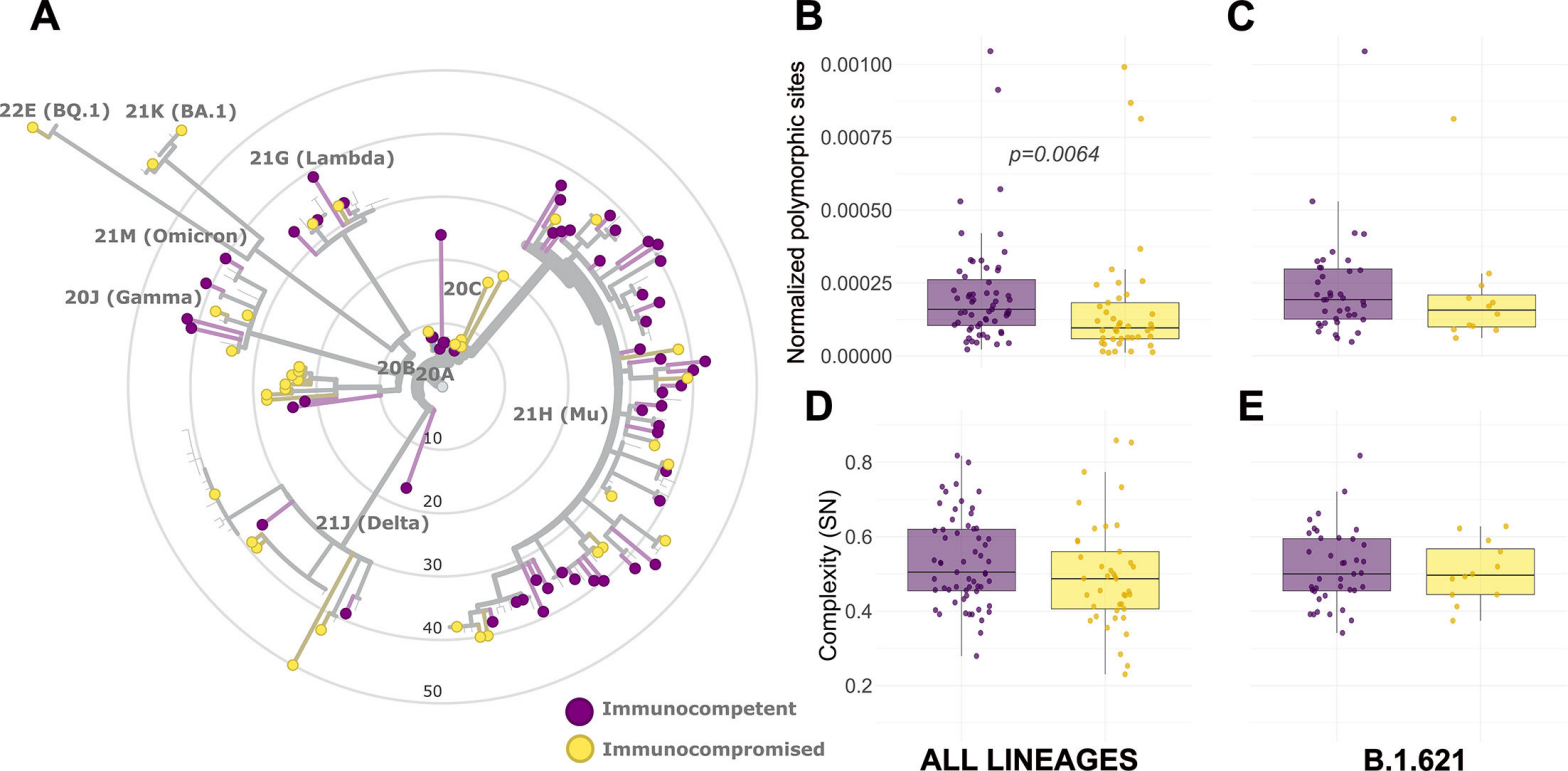
Phylogeny and diversity metrics by immune status. (**A**) Maximum-likelihood phylogeny of consensus SARS-CoV-2 genomes from immunocompetent (purple) and immunocompromised (yellow) patients. Major lineages are annotated using Pango/Nextstrain labels, including 21K (BA.1), 22E (BQ.1), 21M (Omicron), 20J (Gamma), 21H (Mµ), 21J (Delta), and 21G (Lambda). The scale bar indicates substitutions per site. (B, C) Boxplots of normalized polymorphic sites comparing immunocompetent and immunocompromised groups across all lineages (**B**) and within Mµ (B.1.621) (**C**). (D, E) Boxplots of within-host complexity (Shannon entropy of iSNVs) across all lineages (**D**) and within Mµ (B.1.621) (**E**). Points represent individual samples; boxes show median and interquartile range (IQR); whiskers denote 1.5× IQR. Group comparisons in panels B–E were performed using two-sided Wilcoxon rank-sum tests.

Maximum-likelihood phylogenetic analysis confirmed Mµ (B.1.621) as the predominant lineage in the study population, consistent with local circulation during the sampling period (Fig. 2A). Across all lineages, the number of polymorphic sites normalized by mean sequencing depth differed significantly by immune status, with higher values observed in immunocompetent than immunocompromised individuals (two-sided Wilcoxon rank-sum test, *P* = 0.0064; Fig. 2B). In contrast, within-host complexity (Shannon index, Fig. 2D) and Hamming distance (Fig. S2) did not differ between immune groups when all lineages were pooled. Restricting the analysis to Mµ (B.1.621) yielded no between-group differences in the normalized polymorphic sites (Fig. 2C), Shannon index (Fig. 2E), or Hamming distance (Fig. S2). Similarly, neither the number of iSNVs per sample nor allele-frequency distributions differed by immune status, whether considering all lineages or the Mµ subset (Fig. S3). Collectively, these results indicate that, in our study groups, immune status was associated with a modest difference in normalized polymorphic sites when lineages were pooled, while iSNV-based diversity metrics were broadly comparable between groups during acute infection.

### Immune status-stratified iSNVs by genomic region

**Fig 3:**
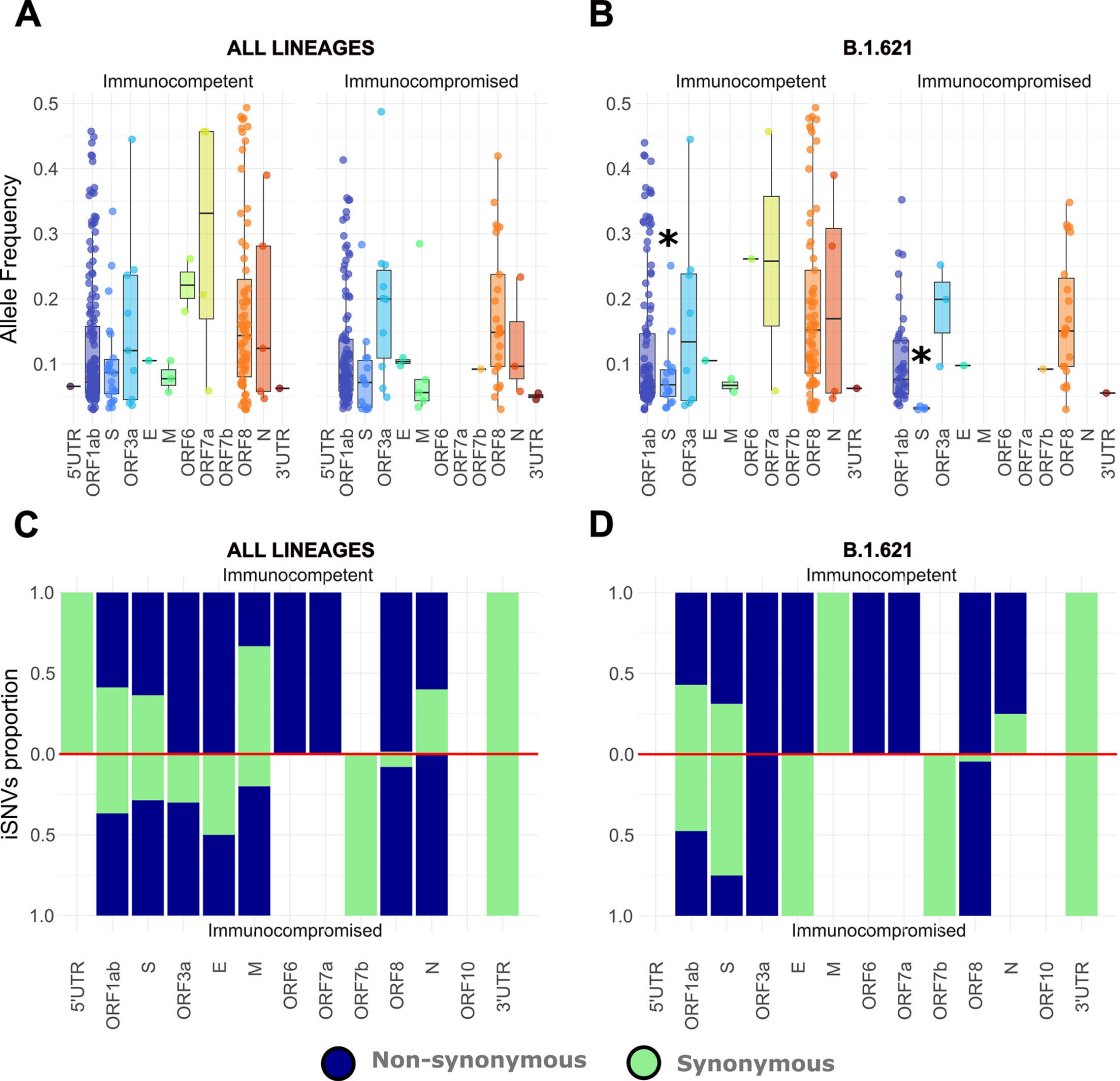
Allelic frequency and the proportion of synonymous and non-synonymous SARS-CoV-2 mutations across genomic regions by immune status. (A, B) Distributions of allelic frequencies for iSNVs across genomic regions in immunocompetent and immunocompromised individuals. Results are shown for all lineages combined (**A**) and for the Mµ variant (B.1.621) (**B**). Each dot represents an iSNV detected within the corresponding genomic region. (C, D) Proportion of synonymous (light green) and non-synonymous (blue) iSNVs across genomic regions in immunocompetent and immunocompromised individuals, shown for all lineages (**C**) and for the Mµ variant (B.1.621) (**D**). The red horizontal line is a visual divider: bars above the line correspond to immunocompetent individuals, and bars below the line correspond to immunocompromised individuals.

A total of 455 iSNVs were identified, of which 281 were attributed to immunocompetent patients and 174 to immunocompromised patients (Table S3). To assess immune status-related differences in iSNVs by genomic region, we compared allele-frequency distributions and the proportion of synonymous versus non-synonymous mutations between immunocompetent and immunocompromised groups across all lineages and then restricted to Mµ (B.1.621) (Fig. 3). Across all lineages, no significant between-group differences in allele-frequency distributions were detected for any genomic region (Fig. 3A). In contrast, when the analysis was restricted to Mµ (B.1.621), higher allele frequencies in the S region were observed in immunocompetent versus immunocompromised individuals (two-sided Wilcoxon rank-sum test, *P* = 0.0016; Fig. 3B).

Regarding coding effect, S-region iSNVs were predominantly non-synonymous in both groups when all lineages were pooled (Fig. 3C). Within Mµ, S-region iSNVs in immunocompromised individuals were mostly synonymous, whereas non-synonymous iSNVs predominated in immunocompetent individuals (Fig. 3D). Taken together, these results show no immune status-related differences in allele-frequency distributions when lineages are pooled, but a Mµ-specific difference confined to the S region, alongside distinct synonymous/non-synonymous profiles by immune status.

### Recurrent iSNVs by immune status across all lineages and within Mµ (B.1.621)

**Fig 4:**
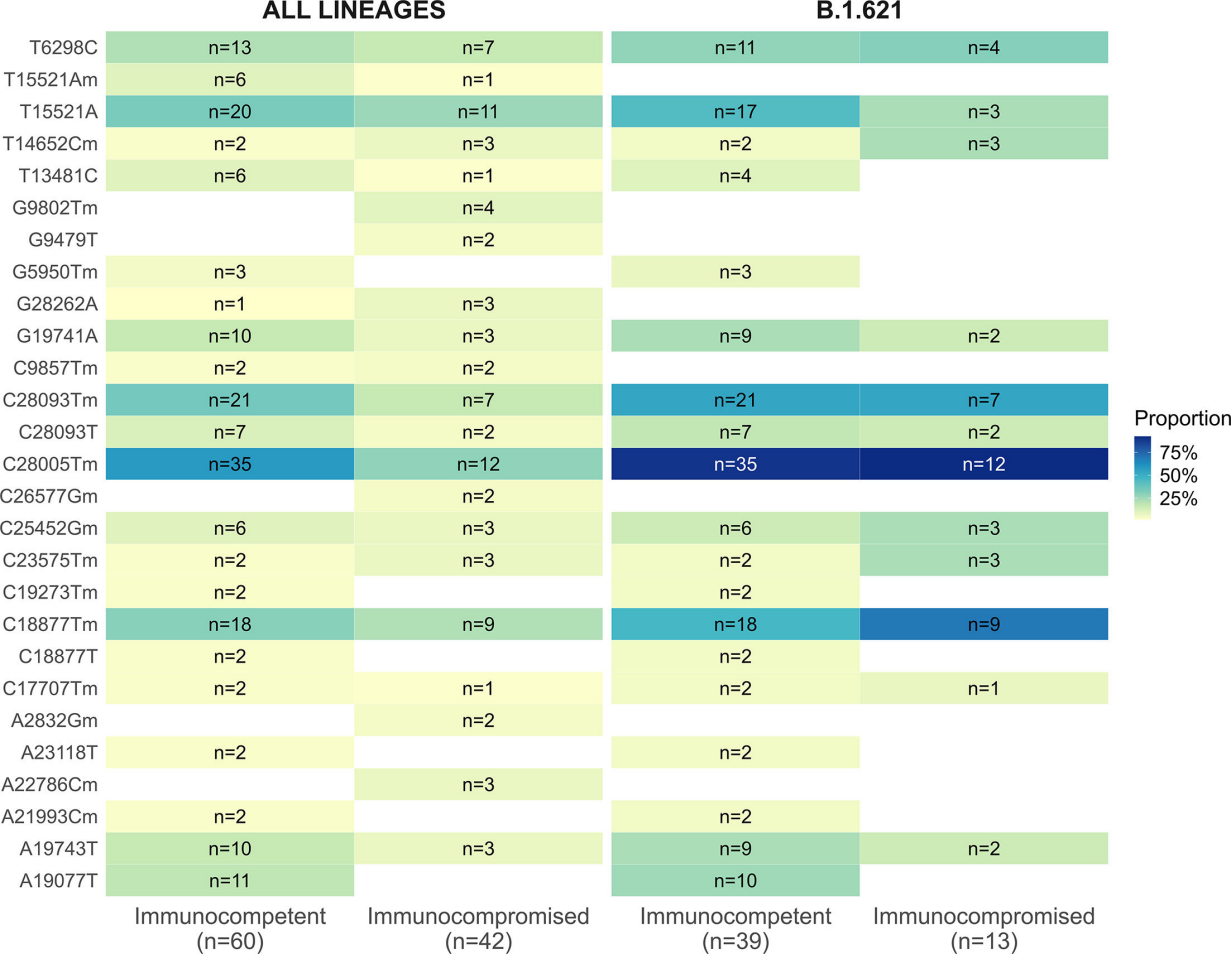
Heatmap of recurrent iSNVs in immunocompetent and immunocompromised individuals across all SARS-CoV-2 lineages (left panel) and within the Mµ variant (B.1.621) (right panel). Each row corresponds to an iSNV meeting the recurrence threshold (observed in ≥2 individuals in at least one immune-status group). Columns indicate immune-status groups; total sample sizes are shown on the *x*-axis. Color intensity represents the proportion of individuals in each group carrying the corresponding iSNV. White cells indicate absence of the iSNV in that group. Mutations ending with “m” denote reversions.

Using a recurrence threshold of ≥2 individuals per group, a shared set of recurrent iSNVs was observed across both immune status groups and in both Fig. 4 panels (reversions noted through suffix “m”): C28005Tm, C28093Tm, C18877Tm, T15521A, T69298C, G19741A, C28093T, C25452Gm, and A19743T. Within the all-lineages panel, C28005Tm was the most frequent event among immunocompetent individuals. When considering all lineages, several iSNVs were detected exclusively in one immune group. Among immunocompromised patients: G9802Tm, G9479T, C26577Gm, and A22786Cm. Among immunocompetent patients: G5950Tm, C19273Tm, C18877T, A23118T, A21993Cm, and A19077T.

Restricting the analysis to Mµ (B.1.621), no iSNV met the recurrence criterion exclusively in immunocompromised patients. In contrast, the following were exclusive to immunocompetent patients within Mµ: T13481C, G5950Tm, C19273Tm, C18877T, A23118T, A21993Cm, and A19077T. Several of these (e.g., G5950Tm, C19273Tm, C18877T, A23118T, A21993Cm, A19077T) also appeared as immunocompetent-exclusive in the all-lineages panel, indicating consistency of the pattern when restricting to the dominant lineage. Taken together, data show a shared core of recurrent iSNVs across immune groups, alongside group-specific recurrent events that are evident when all lineages are pooled and remain detectable within Mµ chiefly among immunocompetent patients under the applied recurrence threshold.

## DISCUSSION

We investigated intra-host SARS-CoV-2 diversity in immunocompromised and immunocompetent individuals during the acute phase of infection, focusing on minor allelic variants (iSNVs). The study population, sampled in Cali, Colombia during a period of Mµ (B.1.621) predominance, provides a snapshot of early within-host viral diversity across immune states. Consistent with prior studies, most iSNVs occurred at low allele frequencies, reflecting strong transmission and within-host bottlenecks during acute infection, including among immunocompromised hosts (2, 3). At the population level, overall iSNV burdens and allele-frequency distributions were comparable between immunocompromised and immunocompetent individuals; however, immunocompetent individuals showed a higher normalized polymorphic sites than immunocompromised individuals across all lineages (*P* = 0.0064). When analyses were restricted to Mµ (B.1.621), a significant difference emerged confined to the S region: immunocompetent individuals exhibited higher S-region iSNV allele frequencies than immunocompromised individuals, a pattern reminiscent of observations reported in vaccinated populations (11).

Mutational patterns also differed by lineage and immune status. When all lineages were pooled, S-region iSNVs were predominantly non-synonymous in both groups. In contrast, within Mµ (B.1.621), S-region iSNVs were mostly synonymous among immunocompromised individuals, whereas non-synonymous changes predominated in immunocompetent hosts. These findings underscore the value of combining pooled and lineage-specific analyses to distinguish effects shared across lineages from lineage-restricted variation.

Although iSNVs are sub-consensus and functional consequences cannot be inferred directly from this data set, synonymous changes are not necessarily neutral; they can influence RNA secondary structure, codon usage and translation kinetics, and interactions with RNA-binding elements (27–30). Accordingly, the higher proportion of synonymous S-region iSNVs in immunocompromised individuals within Mµ highlights the importance of reporting mutation class alongside allele frequencies and motivates functional follow-up (e.g., RNA structure modeling, codon-usage metrics, or deep-mutational scanning) to determine whether these synonymous variants modulate viral gene expression or replication early in the infection. Notably, the synonymous evolutionary rate within the S region has been reported to be low and primarily localized to partly constrained regions (28). Moreover, convergence of viral codon usage toward human-preferred codons can competitively reallocate tRNA pools, thereby attenuating host protein synthesis and prioritizing viral protein production (30).

Stratification also revealed patterns in recurrent iSNVs. Several sites were shared across immune groups, and a subset appeared group-specific when all lineages were pooled; within Mµ, no recurrent iSNV met the exclusivity criterion in immunocompromised patients, whereas several did in immunocompetent patients. Among the recurrent iSNVs detected across all lineages and within Mµ (B.1.621), the set enriched in the immunocompetent group—G5950Tm, C19273Tm, A21993Cm, C18877T, A23118T, and A19077T, comprised three reversions (suffix “m”) and three forward substitutions (two A→T and one C→T). All of these sites have been reported in GISAID since early 2020 (data accessed 28 October 2025); several continued to be deposited through 2025 (G5950T, C19273T, C18877T, A21993C, A19077T), whereas A23118T was reported from 2020 through September 2024. These changes are not Mµ-specific; their sustained detection over multiple years is consistent with recurrent occurrence and persistence in the broader population.

This study has limitations. Its cross-sectional design precludes tracking the emergence, persistence, or fixation of iSNVs over time. Some lineage-by-group strata were small, reducing power for stratified comparisons. Immune status was abstracted from clinical records, so misclassification cannot be excluded (e.g., unrecognized immunocompromising conditions in the immunocompetent group). In addition, corresponding serum samples were not available; therefore, we could not assess binding or neutralizing antibody responses, which might have provided further insight into the relationship between host immune pressure and within-host viral diversity. Finally, iSNV inferences depend on coverage, calling thresholds, and depth normalization; residual technical variability may influence low-frequency calls. Temporal distributions also differed somewhat between immune groups across the study period; therefore, pooled all-lineage analyses should be interpreted cautiously, as they were intended to assess patterns shared across lineages, including recurrent iSNVs observed over time, rather than lineage-specific iSNVs.

In summary, immunocompromised status did not universally associate with greater intra-host diversity during the acute infection stage in this study population. Lineage-specific analyses revealed an Mµ-restricted difference in the S region and a mutational shift in which immunocompromised hosts carried more synonymous S-region iSNVs, whereas immunocompetent hosts showed more non-synonymous S-region iSNVs. These results support reporting pooled and lineage-restricted analyses together and underscore the importance of documenting synonymous iSNVs when characterizing within-host SARS-CoV-2 diversity. Future work should prioritize functional validation of synonymous iSNVs, integration of host immune parameters (e.g., B- and T-cell function and innate immunity), and evaluation of how synonymous iSNVs contribute to viral persistence and transmission risk early during the infection.

## Data Availability

The raw whole-genome sequencing reads generated in this study have been deposited in the NCBI Sequence Read Archive (SRA) under BioProject accession PRJNA1393062.

## SUPPLEMENTAL MATERIAL

Supplemental figures jvi.02224-25-s0001.docx Fig. S1 to S3; descriptive legends for Tables S1 to S3.

Supplemental tables jvi.02224-25-s0002.xlsx Tables S1 to S3.

ASM does not own the copyrights to Supplemental Material that may be linked to, or accessed through, an article. The authors have granted ASM a non-exclusive, world-wide license to publish the Supplemental Material files. Please contact the corresponding author directly for reuse.
