# Supplementary material for: Within-host SARS-CoV-2 diversity in immunocompromised patients during acute infection
Source: J Virol. 2026 Jun 9;100(7):e02224-25. doi: 10.1128/jvi.02224-25 (PMC13386825; doi:10.1128/jvi.02224-25)
Supplement: Supplemental figures — Fig. S1 to S3; descriptive legends for Tables S1 to S3. [file jvi.02224-25-s0001.docx]

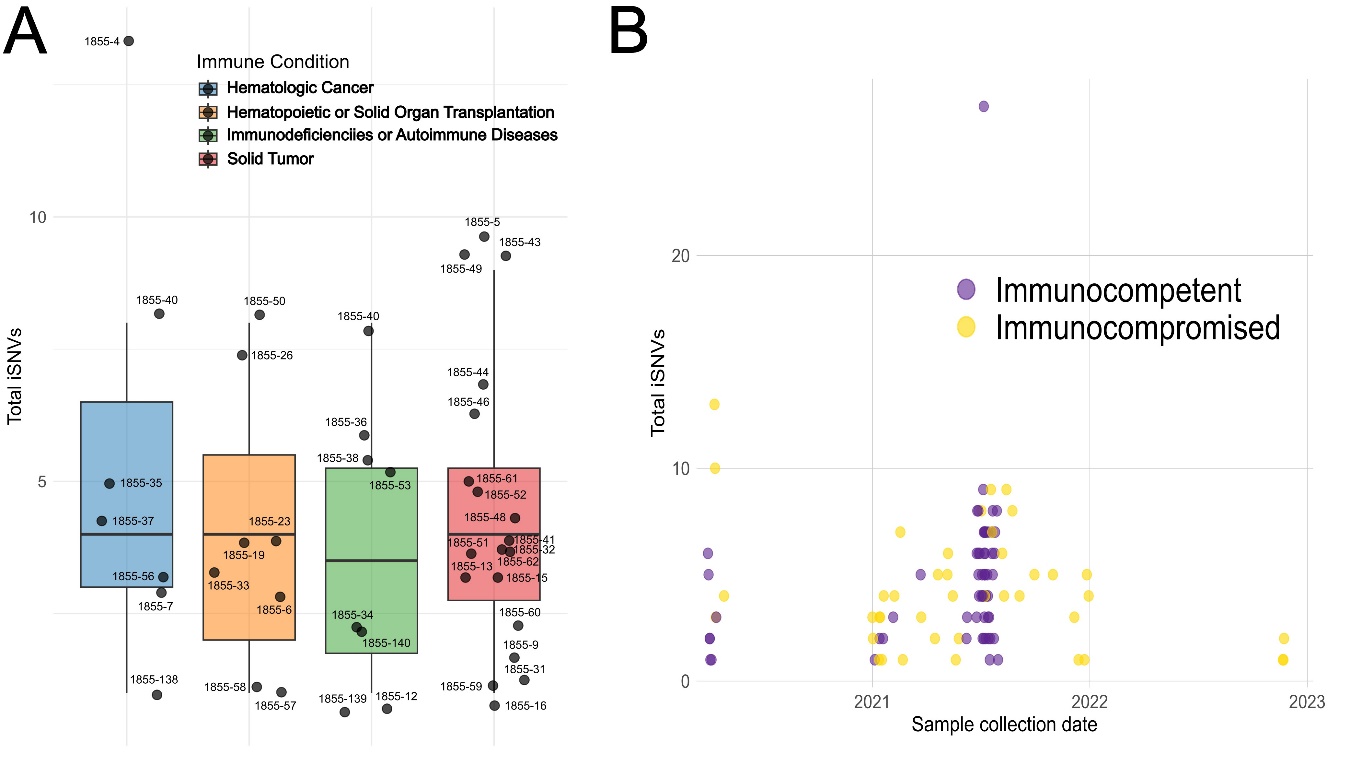


**Figure S1.** *Distribution of intra-host SARS-CoV-2 diversity by immunocompromising condition and over time.* **(A)** Boxplots showing the distribution of total intra-host single nucleotide variants (iSNVs) across different categories of immunocompromising conditions, including hematologic cancer, hematopoietic or solid organ transplantation, primary or secondary immunodeficiencies or autoimmune diseases, and solid tumors. Each dot represents an individual patient, labeled by patient ID. **(B)** Scatter plot of total iSNVs over time according to sample collection date, stratified by immune status (immunocompetent vs. immunocompromised). Each point represents an individual sample. This panel illustrates the temporal distribution of iSNVs across the study period.


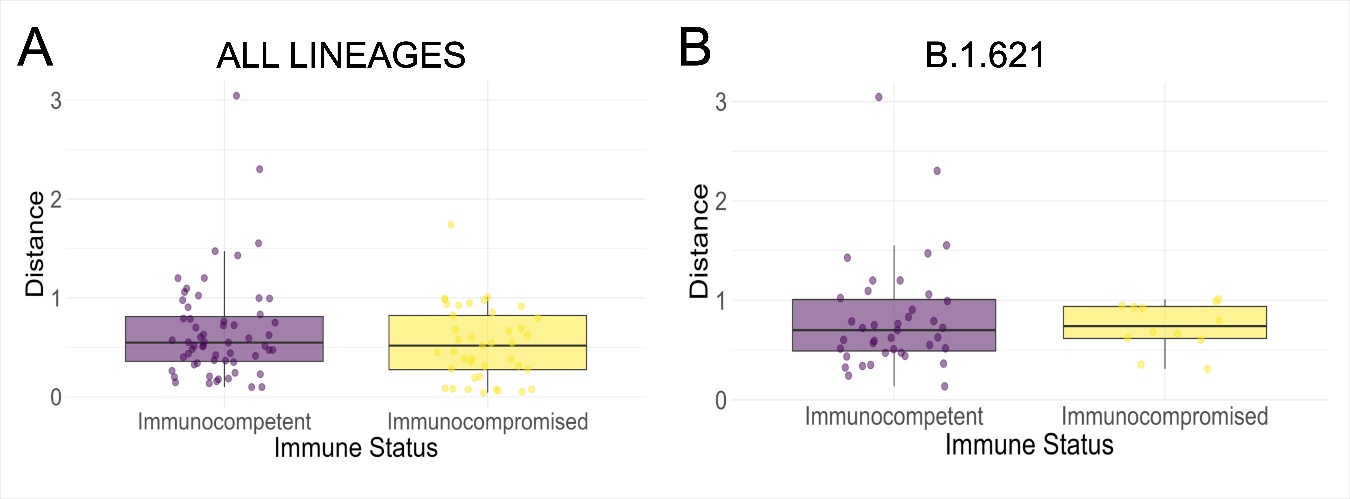


**Figure S2.** *Hamming distance across SARS-CoV-2 genomes by immune status.* **(A)** Boxplots showing the distribution of Hamming distance across all SARS-CoV-2 lineages in immunocompetent and immunocompromised patients. **(B)** Boxplots showing the distribution of Hamming distance for the Mµ variant (B.1.621) stratified by immune status. Hamming distance was calculated as the sum of iSNV frequencies within each sample, representing the divergence from the consensus sequence. Each dot represents an individual sample.

**
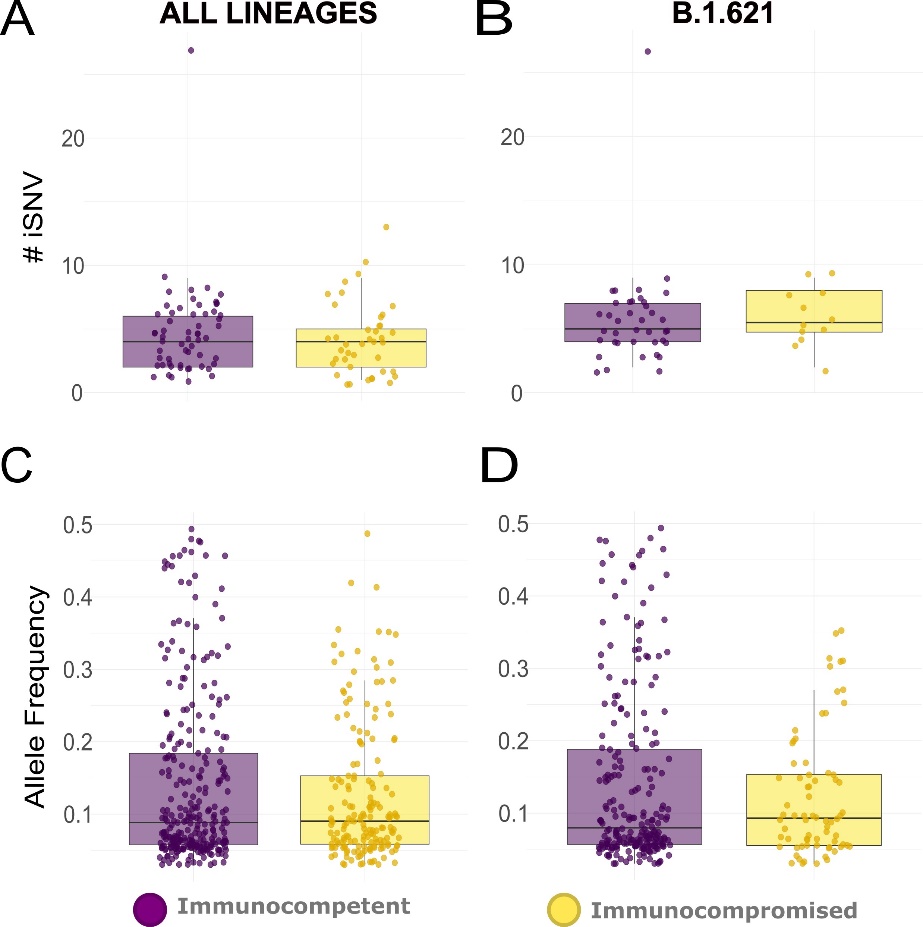
**

**Figure S3.** *Total iSNV burden and allele-frequency distributions by immune status across all lineages and within Mµ (B.1.621)*. (**A–B**) Box-and-whisker plots of the total number of intra-host single-nucleotide variants (iSNVs) per genome in immunocompetent (purple) and immunocompromised (yellow) individuals. Panel **A** shows all lineages combined; Panel **B** is restricted to the Mµ lineage (B.1.621). Each dot represents one genome (sample). (**C–D**) Distribution of iSNV allele frequencies in the same groups. Panel **C** shows all lineages; Panel **D** shows Mµ (B.1.621). Each dot represents one iSNV. Boxes denote the interquartile range (IQR) with the median as the horizontal line; whiskers indicate 1.5×IQR. Colors indicate immune status as in the legend.

**Supplementary table 1**. Clinical, demographic, and laboratory metadata for all enrolled participants, including study ID, demographics, vaccination status, exposure history, sampling dates, symptoms (presence, types, duration), care outcomes (hospitalization/ICU/ventilation), WHO disease severity, comorbidities, immunocompromised status and category, ICD-10 codes, cancer type (if applicable), discharge status, RT-qPCR result/Ct, and assigned SARS-CoV-2 variant/lineage. A compact variable list and data dictionary are provided in Worksheet/Tab 2 of the same Excel file.

**Supplementary table 2**. *Clinical characteristics, immunocompromising conditions, and treatment history of study participants.* This table summarizes demographic variables, underlying immunocompromising conditions, clinical outcomes, the number of intra-host single nucleotide variants (iSNVs) detected per patient, and available treatment history for each participant included in the study. Information on relevant therapies was extracted from clinical records when available, including their timing in relation to SARS-CoV-2 sample collection. Treatment data were not uniformly available for all patients, as some care was provided outside the recruiting institution and external clinical records were not accessible.

**Supplementary table 3**. Per-sample list of all intra-host single-nucleotide variants (iSNVs) that passed quality filters and were retained for analysis. Calls were generated with iVar and required depth ≥400 reads, Phred quality ≥20, and allele frequency 0.03–0.97. The table reports genomic coordinates and alleles (REFERENCE: MN908947.3), per-allele depths/qualities, allele frequency, total depth, pass flag, and functional annotation, including gene/feature, codon and amino-acid context, and amino-acid position. A compact variable list and data dictionary are provided in Worksheet/Tab 5 of the same Excel file.
